# Association of day-case rates with post COVID-19 recovery of elective laparoscopic cholecystectomy activity across England

**DOI:** 10.1308/rcsann.2023.0111

**Published:** 2024-04-02

**Authors:** FM Ayyaz, J Joyner, M Cheetham, TWR Briggs, WK Gray

**Affiliations:** ^1^ Getting It Right First Time Programme, NHS England and NHS Improvement, UK; ^2^ Manchester University NHS Foundation Trust, UK; ^3^ Croydon Health Services NHS Trust, UK; ^4^ The Shrewsbury and Telford Hospital NHS Trust, UK; ^5^ Royal National Orthopaedic Hospital NHS Trust, UK

**Keywords:** Laparoscopic cholecystectomy, Day-case surgery, General surgery, COVID-19

## Abstract

**Introduction:**

The aim of this study was to investigate the safety of day-case laparoscopic cholecystectomy, and the association between day-case rates and, post the COVID-19 pandemic, recovery of activity to prepandemic levels for integrated care boards (ICBs) in England.

**Methods:**

This was a retrospective observational study of the Hospital Episodes Statistics (HES) data set. Elective laparoscopic cholecystectomies for the period 1 January 2019 to 31 December 2022 were identified. Activity levels for 2022 were compared with those for the whole of 2019 (baseline). Day-case activity was identified where the length of stay recorded in the HES was zero days.

**Results:**

Data were available for 184,252 patients across the 42 ICBs in England, of which 120,408 (65.3%) were day-case procedures. By December 2022, activity levels for the whole of England had returned to 88.2% of prepandemic levels. The South West region stood out as having recovered activity levels to the greatest extent, with activity at 97.3% of prepandemic levels during 2022. The South West also had the highest postpandemic day-case rate at 74.9% of all patients seen as a day-case during 2022; this compares with an England average of 65.3%. At an ICB level, there was a significant correlation between day-case rates and postpandemic activity levels (*r* = 0.362, *p *= 0.019). There was no strong or consistent evidence that day-case surgery had poorer patient outcomes than inpatient surgery.

**Conclusions:**

Recovery of elective laparoscopic cholecystectomy activity has been better in South West England than in other regions. Increasing day-case rates may be important if ICBs in other regions are to increase activity levels up to and beyond prepandemic levels.

## Introduction

The COVID-19 pandemic has had a significant impact on healthcare systems across the world, with elective surgical activity being disrupted in many countries to accommodate the surge in COVID-19 cases. The situation in England is no exception, as the National Health Service (NHS) was forced to temporarily halt most elective procedures to conserve resources and protect patients and healthcare workers from the spread of the virus.^1^ The resumption of elective surgical activities has been slow and phased, with guidelines being put in place to ensure the safety of patients and healthcare workers.^2^

Laparoscopic cholecystectomy is a common elective surgical procedure that involves removal of the gallbladder. It is performed using minimally invasive techniques through small incisions in the abdomen. The use of minimally invasive surgery can allow faster recovery times and shorter hospital stays than open surgery. As a consequence, day-case laparoscopic cholecystectomy is common, with outcomes at least as good as for open surgery.^3^ However, the COVID-19 pandemic has led to a reduction in the number of laparoscopic cholecystectomies performed, with some patients being forced to wait for up to 12 months for surgery, and this is likely to impact patient outcomes.^4,5^ Delays caused by disruptions in services lead to an increased readmission rate of patients with biliary colic, which predisposes patients to have a difficult cholecystectomy. This, in turn, impairs the ability for these procedures to be done as a day-case.^6^

In view of the above, there is a need to assess the extent of the impact on laparoscopic cholecystectomy day-case rates and patient outcomes in England following the pandemic and to understand the factors affecting their recovery.

The aim of this study was to investigate the association between postpandemic activity levels and day-case rates for laparoscopic cholecystectomies in England.

## Methods

### Study design

This was a retrospective analysis of administrative data from the UK Hospital Episode Statistics (HES) database. The design and analysis framework follows that of a cohort study of exposed and unexposed groups. The HES database is collected by NHS Digital and includes data for NHS hospital activity in England. HES includes procedures funded by the NHS, but conducted by non-NHS providers (independent sector/private hospitals). HES data are mandatory and primarily collected to allow reimbursement.

### Ethics

The presentation of data follows current NHS Digital guidance for use of HES data for research purposes.^7^ Consent from individuals involved in this study was not required for analysis of this administrative data set.

### Setting

All NHS hospitals in England are run by trusts, each of which covers a geographically defined catchment area of varying physical size and population. Trusts together with local government bodies are grouped into integrated care systems with each having an integrated care board (ICB) responsible for the health and care needs of its catchment population. The number of NHS trusts in each ICB varies widely, although most ICBs comprise between three and eight NHS hospital trusts.

### Study period

The study period was 1 January 2019 to 31 December 2022 for the index procedure. Follow-up data were available for all patients for 30 days postsurgery.

### Inclusion and exclusion criteria for index procedure

We identified cholecystectomy using the Office of Population Censuses and Surveys Classification of Interventions and Procedures version 4 (OPCS-4) codes: J181 (total cholecystectomy and excision of surrounding tissue) or J183 (total cholecystectomy not elsewhere classified) used in the first position in the procedural record together with the code Y752 (laparoscopic approach to abdominal cavity not elsewhere classified) used in any position in the procedural record.

Patients were excluded if the procedure was nonelective or the patient was aged <17 years.

### Patient outcomes

1. Emergency hospital readmission within 30 days of discharge with an overnight stay. This outcome was chosen to reflect the need for an early readmission related to a complication of surgery and is an NHS England data standard. Emergency readmissions without an overnight stay were excluded because they were likely to be for more minor issues and such admissions/attendances are recorded variably across hospitals.
2. Likely complications of surgery identified during emergency admission within 30 days postdischarge. These were complications of procedures, not elsewhere classified (ICD-10 code T81) or abdominal or pelvic pain (ICD-10 code R10).
3. Mortality at 30 days postdischarge. Mortality data were taken from the UK Office for National Statistics.

### Operational outcome

The operational outcome was recovery of activity during January to December 2022 relative to baseline (January to December 2019) levels. Activity was measured by the number of laparoscopic cholecystectomies conducted in each period. For modelling, this outcome was dichotomised into ICBs with rates for recovery of activity that were above or below the England average (88.2%).

### Primary exposure variable

The primary exposure variable was day-case or inpatient stay. Day-case was defined as any procedure where the admission day and discharge day were the same. This definition varies from that used by the British Association of Day Surgery; namely, that the operation was planned as a day-case and the patient went home on the same day. The definition used here was preferred because it reflected clinical practice. However, the numbers of planned and actual day-case procedures are reported and the discrepancy between the two definitions is very small.

### Secondary exposure variable

In secondary analysis, the exposure variable was defined at an ICB level; ICBs with a day-case rate above and below the England average (65.3%) were compared. This analysis was conducted to minimise bias when considering the data at a patient level because patients with more severe illness presentation (and so potentially poorer outcomes) are more likely to stay in hospital overnight. We were unable to adjust for severity of presentation directly.

### Covariates

1. Age in years was categorised into six age bands (17–29, 30–39, 40–49, 50–59, 60–69 years and ≥70) for analysis. These age bands were chosen to allow broadly similar number of patients in each group and to ensure the cut-offs were clinically meaningful.
2. Sex.
3. Charlson Comorbidity Index (CCI), a weighted score derived from the presence of 17 comorbidities (peripheral vascular disease, congestive heart failure, acute myocardial infarction, cerebrovascular disease, dementia, chronic pulmonary disease, connective tissue disease/rheumatic disease, peptic ulcer, liver disease [mild and moderate/severe], diabetes [with and without chronic complications], paraplegia/hemiplegia, renal disease, cancer [primary and metastatic] and HIV/AIDS).^8^ The comorbidity was deemed present if it was recorded in the HES data set as a secondary diagnosis in the index admission or as a primary or secondary diagnosis in any admission during the previous year, in accordance with the recommendations of Quan *et al.*^9^ Scores were categorised as 0, 1, 2, 3 and ≥4 for analysis based on the small number of patients with scores >4.
4. Deprivation was recorded using the Index of Multiple Deprivation (IMD) for the Lower Super Output Area (LSOA) of the patients’ home address. The Office for National Statistics compile the IMD score based on a number of markers for deprivations; we used the latest updated values from 2019. Scores are recorded for each of the 32,844 LSOAs in England at the time of the 2011 census. For analysis, scores were categorised into quintiles based on England averages.
5. Postprocedural complications (ICD-10 code T81) identified during the index stay. This was included as a covariate because postprocedural complications identified during the index stay could preclude discharge on the day of surgery. The code for abdominal or pelvic pain was not used as a covariate because it would be ambiguous whether the pain was pre- or postprocedural.
6. Diagnoses recorded on admission. ICD-10 code K800 was used to identify patients with calculus of gallbladder with acute cholecystitis, ICD-10 codes K801 (calculus of gallbladder with other cholecystitis) and K804 (calculus of bile duct with cholecystitis) were used to identify patients with calculi with nonacute cholecystitis. ICD-10 codes K802 (calculus of gallbladder without cholecystitis), K803 (calculus of bile duct with cholangitis), K805 (calculus of bile duct without cholangitis or cholecystitis) and K808 (other cholelithiasis) were used to identify calculi without cholecystitis. ICD-10 code K810 was used to identify patients with acute cholecystitis without calculi. ICD-10 codes K811 (chronic cholecystitis), K818 (other cholecystitis) and K819 (cholecystitis unspecified) were used to identify nonacute cholecystitis without calculi.

### Data management and statistical analyses

Data were analysed using standard statistical software, namely Microsoft Excel (Microsoft Corp., Redmond, WA, USA), Stata (Stata Corp LLC, College Station, TX, USA) and Alteryx (Alteryx Inc., Irvine, CA, USA).

All data were categorical and were summarised using frequency and percentage. Owing to a relatively large proportion of procedures taking place in non-NHS providers, patients were grouped into ICBs based on the patient’s LSOA of residence. This avoided a potentially confounding effect due to non-NHS providers having a less complex case-mix. Multivariable logistic regression models were constructed for the outcomes described above and using the listed exposure variables and covariates (age, sex, comorbidity, deprivation and index postprocedural complications). Model fit was assessed with reference to eigenvalues, tolerance and by examination of residual plots. Confidence intervals (CIs) were used to draw inference, with a 95% CI for an odds ratio (OR) not including the value 1 taken to indicate significance.

## Results

A total of 184,252 elective laparoscopic cholecystectomies were identified over the four-year study period, of which 120,408 (65.3%) were day-case procedures and 63,844 (34.7%) involved an inpatient stay.

Of the 184,252 procedures, intended management prior to admission was recorded for 178,696 (97.0%) patients. Of these, 116,348 (65.1%) were conducted as day-case and 62,348 (34.9%) as inpatient stay. Of the 116,348 day-case procedures, day-case was planned in 109,955 (94.5%). For 62,348 with an inpatient stay, only 22,498 (36.1%) were planned as inpatient stay, with the remaining 39,850 (63.9%) converted from day-case to inpatient stay following admission.

### Outcomes for day-case and inpatient surgery

The profile of patients and their outcomes according to whether they had day-case or inpatient stay surgery are summarised in Table 1. Patients undergoing surgery as inpatients were generally older, more likely to be male and have a greater number of comorbidities. Diagnoses recorded on admission were similar, with the most obvious difference being that patients with acute cholecystitis were approximately twice as likely to be operated on as inpatients than as day-case patients. All outcomes studied were poorer for inpatients, at least partly reflecting their demographic profile. Table 2 summarises the outputs for the multivariable logistic regression models comparing outcomes for day-case and inpatient surgery. Comparing day-case patients and inpatients directly, 30-day emergency readmission, 30-day mortality and postprocedural complications were significantly less common in the day-case surgery group even after adjusting for age, sex, comorbidity, deprivation and index complications. When ICBs with above and below England average day-case rates were compared, there was no difference between the two groups for 30-day emergency readmission or 30-day mortality. Emergency readmission with postprocedural complications was significantly more common in ICBs with high day-case rates, although the increase in odds was very modest (OR 1.05, 95% CI 1.00 to 1.10; *p *= 0.036).

**Table 1:** Profile of all patients undergoing elective laparoscopic cholecystectomy

Variable	Day-case (*N *= 120,408)	Inpatient stay (*N* = 63,844)
Demographic factors, *n* (%)		
Age band (years)		
17–29	13,854 (11.5)	4,289 (6.7)
30–39	23,162 (19.2)	8,535 (13.4)
40–49	22,890 (19.0)	9,903 (15.5)
50–59	27,612 (22.9)	13,754 (21.5)
60–69	19,729 (16.4)	12,822 (20.1)
70 and over	13,161 (10.9)	14,541 (22.8)
Sex (22 missing values)		
Female	91,723 (76.2)	44,795 (70.2)
Male	28,669 (23.8)	19,043 (29.8)
Charlson Comorbidity Index		
0	78,581 (65.3)	33,549 (52.5)
1	30,214 (25.1)	17,900 (28.0)
2	7,828 (6.5)	6,760 (10.6)
3	2,366 (2.0)	2,929 (4.6)
≥4	1,419 (1.2)	2,706 (4.2)
Deprivation (1,703 missing values)		
1 (most deprived)	26,421 (22.1)	14,047 (22.2)
2	25,251 (21.2)	13,194 (20.9)
3	24,273 (20.3)	12,719 (20.1)
4	22,879 (19.2)	12,126 (19.2)
5 (least deprived)	20,545 (17.2)	11,094 (17.6)
Diagnosis on admission		
Calculi with acute cholecystitis	4,903 (4.1)	5,241 (8.2)
Calculi with nonacute cholecystitis	63,627 (52.8)	33,251 (52.1)
Calculi without cholecystitis	36,296 (30.1)	15,693 (24.6)
Acute cholecystitis without calculi	932 (0.8)	1,208 (1.9)
Nonacute cholecystitis without calculi	10,031 (8.3)	5,967 (9.3)
Other diagnosis	4,619 (3.8)	2,484 (3.9)
Outcomes of the index stay		
Complication recorded during index stay	1,255 (1.0)	2,149 (3.4)
Postdischarge outcomes		
Emergency hospital readmission within 30 days of discharge	4,742 (3.9)	3,698 (5.8)
Death within 30 days of discharge	33 (0.03)	70 (0.1)
Complication recorded on emergency readmission within 30 days of discharge	5,007 (4.2)	3,157 (4.9)

**Table 2:** Summary of multivariable models of the relationship between day-case surgery and outcomes

	Odds ratio (95% confidence interval)	
	Direct comparison of day-case and inpatients	Comparison of ICBs with high and low day-case rates
Emergency readmission within 30 days of discharge	0.72 (0.69 to 0.76) *p *< 0.001	1.04 (1.00 to 1.09) *p *= 0.082
Complication recorded within 30 days of discharge	0.81 (0.77 to 0.85) *p *< 0.001	1.05 (1.00 to 1.10) *p *= 0.036
Death within 30 days of discharge	0.57 (0.37 to 0.88) *p *= 0.012	0.95 (0.64 to 1.41) *p *= 0.798

Note: An odds ratio <1 indicates that the outcome was less common for patients undergoing day-case surgery/operated on at an ICB with a high day-case rate and an odds ratio >1 indicates that the outcome was more common for patients undergoing day-case surgery/operated on at an ICB with a high day-case rate. Models were adjusted for age band, sex, deprivation, Charlson Comorbidity Index score and index postprocedural complications.

ICB = integrated care board

**Figure 1:**
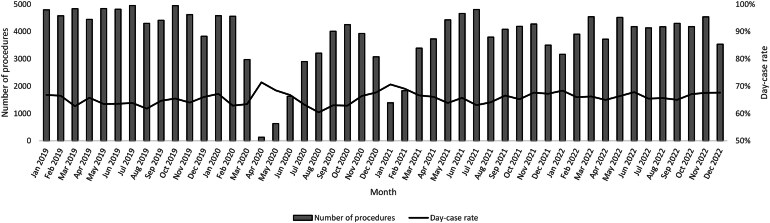
Activity levels and day-case rates per month for laparoscopic cholecystectomy (January 2019 to December 2022)

Table 3 presents data on patient numbers, patient profiles, day-case rates and outcomes across the four-year study period and Figure 1 presents data on patient numbers and day-case rates for each month of the study. For 2022, patient numbers were 88.2% of those for 2019. The proportion of older patients and their diagnoses were similar, although there was a small increase in the proportion of patients with multiple comorbidities across the four years. Despite large fluctuations in patient numbers due to the suspension of elective activity in England during the first and second waves of the COVID-19 pandemic, day-case rates remained stable over the four years. Likewise, outcomes have remained relatively unchanged over time. Although there has been a small increase in the absolute proportion of complications recorded during the index stay this has been counterbalanced by a fall in recorded complication rates postdischarge. Despite the effects of the COVID-19 pandemic, 30-day readmission rates fell slightly over the study period.

**Table 3:** Change in proportion of patients operated on as day-case and outcomes over the study period

	Financial year			
	2019	2020	2021	2022
Number of patients	55,373	35,883	44,094	48,902
Aged ≥60 years	18,151 (32.8)	11,579 (32.3)	14,168 (32.1)	16,355 (33.4)
Charlson Comorbidity Index ≥2	6,874 (12.4)	4,422 (12.3)	5,800 (13.2)	6,912 (14.1)
Region of residence (1,703 missing values)				
East of England	6,478 (11.8)	4,230 (11.9)	5,125 (11.7)	5,833 (12.0)
London	6,277 (11.4)	4,183 (11.8)	5,092 (11.6)	5,699 (11.8)
Midlands	10,858 (19.8)	6,113 (17.2)	8,337 (19.1)	9,243 (19.1)
North East and Yorkshire	9,905 (18.0)	6,343 (17.9)	8,031 (18.4)	9,019 (18.6)
North West	7,361 (13.4)	4,491 (12.7)	5,725 (13.1)	6,234 (12.9)
South East	8,295 (15.1)	5,707 (16.1)	6,560 (15.0)	6,797 (14.0)
South West	5,741 (10.5)	4,405 (12.4)	4,882 (11.2)	5,585 (11.5)
Diagnosis on admission				
Calculi with acute cholecystitis	2,740 (4.9)	1,970 (5.5)	2,528 (5.7)	2,906 (5.9)
Calculi with nonacute cholecystitis	28,732 (51.9)	18,634 (51.9)	23,370 (53.0)	26,142 (53.5)
Calculi without cholecystitis	16,447 (29.7)	9,703 (27.0)	12,270 (27.8)	13,569 (27.7)
Acute cholecystitis without calculi	658 (1.2)	420 (1.2)	537 (1.2)	525 (1.1)
Nonacute cholecystitis without calculi	4,912 (8.9)	3,407 (9.5)	3,707 (8.4)	3,972 (8.1)
Other diagnosis	1,884 (3.4)	1,749 (4.9)	1,682 (3.8)	1,788 (3.7)
Number of patients as day-case	35,739 (64.5)	23,116 (64.4)	29,037 (65.9)	32,516 (66.5)
Complication recorded during index stay	899 (1.6)	595 (1.7)	841 (1.9)	1,069 (2.2)
Emergency readmission within 30 days of discharge	2,705 (4.9)	1,644 (4.6)	2,025 (4.6)	2,066 (4.2)
Complication recorded on emergency readmission within 30 days of discharge	2,540 (4.6)	1,562 (4.4)	2,011 (4.6)	2,051 (4.2)

Values are given as *n* (%)

### Recovery of activity levels following the COVID-19 pandemic

**Figure 2:**
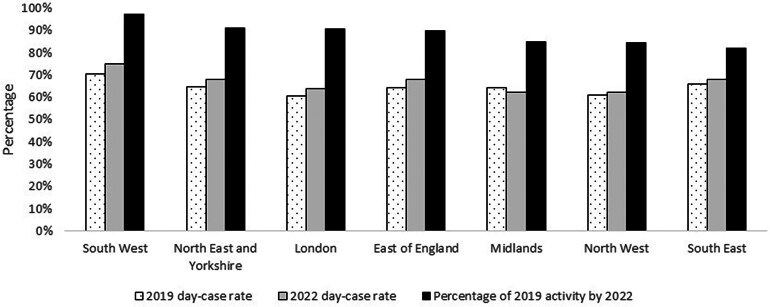
Percentage recovery of 2019 activity levels during 2022 and day-case rates for 2019 and 2022

Figure 2 summarises day-case rates in 2019 and 2022 across the seven NHS England regions. Most regions showed small increases in day-case rates from 2019 to 2022, but there was a fall in day-case rates in the Midlands. Figure 2 also shows the recovery of activity levels during 2022 compared with 2019 for each region. In general, regions with high day-case rates in 2022 tend to have recovered a greater proportion of their 2019 activity levels. The South West had regained 97.3% of its 2019 activity level and had a day-case rate in 2022 of 74.9%. This compares with the Midlands, with a day-case rate of 62.1% in 2022 and a recovery in activity of only 85.1%. One possible exception to this pattern is the South East, which had a relatively high day-case rate (68.0%) in 2022, but a recovery of activity of only 81.9%.

At an ICB level, there was a significant association between day-case rates and recovery of activity postpandemic (*r* = 0.362, *p *= 0.019). Figure S1 (available online) depicts the relationship graphically. A multivariable regression model using data from 2022 comparing only ICBs above and below the England average for recovery of activity from baseline also suggested that day-case surgery was associated with greater recovery of activity (OR = 1.21, 95% CI 1.19 to 1.24; *p *< 0.001) after adjusting for differences in age, sex, comorbidity profile, deprivation and index complications. The full model is summarised in Table S1 (available online).

## Discussion

Our analysis of laparoscopic cholecystectomies performed in England over a four-year period provides evidence that day-case laparoscopic cholecystectomy is safe, and that day-case surgery can support recovery of activity levels following the COVID-19 pandemic. As with all elective surgery in England, elective laparoscopic cholecystectomy procedures were suspended from March 2020.^10^ Although activity recovered during late 2020, elective surgery was partially suspended again in January 2021. The impact of these suspensions on activity levels can be seen in our data. Despite these fluctuations in activity levels, day-case rates, and the profile of patients undergoing surgery, remained remarkably stable over the four years studied.

Day-case cholecystectomy is not suitable for all. Several studies have investigated the safety, acceptability and feasibility of day-case laparoscopic cholecystectomy for selected low-risk patients, although sample sizes tend to be small and from single sites.^11–15^ A recent study in Tanzania showed that 75.2% of 109 planned day-case laparoscopic cholecystectomies were conducted successfully as day-cases and advocated the setting up of day-case units for this purpose.^15^ The greater efficiency of day-case surgery may be especially relevant to low- and middle-income countries, where resources may be particularly limited.

A systematic review published in 2013 by Vaughan *et al* identified six randomised clinical trials of day-case versus inpatient-stay laparoscopic cholecystectomy for review.^14^ The studies comprised a total of 492 patients. There was no significant difference between the two groups in rates of serious adverse events, quality of life, postprocedural pain, return to work, return to activities or hospital readmissions. The authors noted the need for further, more robust studies.

Vaughan *et al* note that there was little evidence that day-case laparoscopic cholecystectomy was associated with better patient outcomes, and authors should avoid claims that day-case surgery is beneficial to patients.^14^ Although outcomes were significantly better when day-case patients and inpatients were compared directly in our study, these findings are likely confounded by severity of presentation and other variables not adjusted for in the modelling. The comparison of ICBs with high and low rates of day-case surgery may be more informative in this regard; in this case, there were no differences in 30-day emergency readmissions and 30-day mortality between the two groups. Although there was a modest increase in the odds of postdischarge emergency readmission with postprocedural complications, this may partly reflect the lower rates of postprocedural complications identified during the index stay for day-case patients, rather than higher overall rates of complications. This finding emphasises the need for day-case surgery to be viewed as a pathway, with patient support during the postdischarge phase. Patients should be supported by community services and should feel fully informed as to who to contact in the event of any postdischarge complications. As such, we support the conclusions of Vaughan *et al*. The key benefit of day-case surgery lies in its increased efficiency, reduced financial cost and reduced carbon footprint.^16–18^

Regions with higher day-case rates tended to recover a greater proportion of their prepandemic activity levels, suggesting the importance of promoting day-case procedures in postpandemic recovery efforts. The South West region had the highest proportion of recovery of activity and had the highest day-case rate. The South West also had the smallest number of patients hospitalised with COVID-19 during the pandemic and the lowest in-hospital mortality rate.^19,20^ Although this may have meant recovery of elective activity was faster in the South West and could have confounded our findings to an extent, the association between day-case rates and recovery of activity was also apparent at an ICB level, suggesting the association was not confined to the South West. A range of factors are likely to be important in supporting trusts to increase day-case rates, and these will vary from trust to trust. However, access to day-case infrastructure (e.g. through NHS England’s surgical hubs programme^21^), a willingness to conduct such procedures as day-case, management support and an in-house clinical champion are likely to be important. Nevertheless, the relatively high day-case rate but low activity recovery rate in the South East indicates that factors other than day-case surgery are important. Further investigation of the factors associated with post-COVID-19 recovery of elective activity is merited.

### Strengths and limitations

Our study had a relatively large sample size, covering four years of elective laparoscopic cholecystectomies in England. The data set is as complete a record as exists for this period and so issues around collider bias should be minimised.^22^ Nevertheless, we caution against generalising our findings to other settings without due care. HES data allow us to follow patient encounters across all hospitals in England, meaning all emergency readmissions should have been captured, regardless of the readmitting hospital.

Our study has a number of limitations, some of which have already been acknowledged. The retrospective design of the study limits the ability to establish causality. There is a need for further trials on the safety of day-case laparoscopic cholecystectomy and study of factors associated with elective recovery. Concerns regarding the consistency of the HES data set have been identified.^23,24^ However, these are often related to the quality of clinical coding. Because laparoscopic cholecystectomy has a relatively simple coding structure, this may be less of a concern for our study. We are not aware of any evidence of systematic bias in data quality between individual NHS trusts or ICBs of a nature that would substantially impact our findings.

## Conclusion

We present evidence of the safety of day-case laparoscopic cholecystectomy. Increasing day-case rates may help to increase activity levels and help reduce waiting times for elective surgery in England.

## Author contributions

This study was designed and organised by FMA, WKG and TWRB. Data cleaning and analysis was by WKG. Writing of the first draft was by FMA and WKG. All authors critically reviewed the manuscript and agreed to submission of the final draft.

## Data Availability

This report does not contain patient-identifiable data. Data in this report are anonymised. The underlying HES data cannot be made available directly by the authors because the data were obtained under licence/data sharing agreement from NHS Digital. HES data are available from NHS Digital upon application.
